# Effects of equine-assisted activities on attention and quality of life in children with cerebral palsy in a randomized trial: examining the comorbidity with attention-deficit/hyperactivity disorder

**DOI:** 10.1186/s12887-021-02597-0

**Published:** 2021-03-19

**Authors:** Bomi Ahn, Yoo-Sook Joung, Jeong-Yi Kwon, Dong Ik Lee, Soohwan Oh, Byoung-Uk Kim, Jung Yoon Cha, Ji-Hae Kim, Ji Young Lee, Hye Yeon Shin, Yun Sik Seo

**Affiliations:** 1 Department of Psychiatry, Samsung Medical Center, Sungkyunkwan University, School of Medicine, 115 Irwon-ro, Gangnam-gu, Seoul, 06351 South Korea; 2 Department of Physical and Rehabilitation Medicine, Samsung Medical Center, Sungkyunkwan University School of Medicine, Seoul, South Korea; 3 Samsung Equestrian Team, Bugok dong, Gunposi, Gyeonggido Republic of Korea

**Keywords:** Cerebral palsy, Attention-deficit hyperactivity disorder, Equine-assisted activities

## Abstract

**Background:**

Attention problems and decreased quality of life are frequently accompanied in Cerebral Palsy (CP), which can negatively affect rehabilitation of physical disability. However, the majority of affected children remain untreated in the aspects of attention or psychosocial factors. Equine-Assisted Activities and Therapies (EAAT) use horse as a therapeutic modality including grooming as well as mounted riding activities in which patients exercise and experience mounted stimulation. It is known to help improve attention in children with ADHD, so that it can be an exercise therapy that is expected to improvement of attention as well as rehabilitating effects in CP patients. EAA may be a promising strategy to address the unmet need for CP patients. This study aims to investigate the efficacy of EAA for children with CP, those with both CP and ADHD and confirm the comorbidity between CP and ADHD.

**Methods:**

Forty-six children with cerebral palsy participated in this study. For the exercise group, they participated in a 40-min session twice a week for a 16-week period, while the control group engaged in daily life without any special treatments. Each children individually were assessed on attention and psychological wellbeing at baseline and post-treatment. Comorbidity were identified based on the Diagnostic and Statistical Manual of Mental Disorder 5th edition (DSM-5) and confirmed by Korean Kiddie-Schedule for Affective Disorders and Schizophrenia Present and Lifetime Version (K-SADS-PL).

**Results:**

Perseveration rated using the Conner’s Performance Test (CPT) showed a significant decrease only in the exercise group (*p < .024)*. However, no significant improvement in children’s quality of life was observed after EAA program compared with control group. Among the total participants, fifteen children (31.91%) were diagnosed with ADHD. When conducting an additional analysis with the subsample of CP patients diagnosed with ADHD, the d’, commission error and perseveration showed a significant decrease only in the exercise group. Children with CP and ADHD reported an improvement in quality of life both in exercise and control group, but only in the exercise group social functioning exhibited a significant difference.

**Conclusion:**

The positive effects of the EAA on attention and quality of life were confirmed. Children with CP in the exercise group were more capable to sustain their attention longer. Those with CP and ADHD showed an increase in attention and perceived to have better social skills after receiving 16 weeks of EAA compared to those in the control group. Considering high comorbidity of CP and ADHD, it seems that the EAA program could be the better alternative treatment for CP with attentional problem. The results of this study will contribute to growing evidence for the efficacy of EAA in children especially with CP and ADHD.

**Trial registration:**

This trial was registered on ClinicalTrials.gov (NCT03870893). Registered 26 July 2017.

## Background

Cerebral palsy (CP) is a group of neurological disorders resulting from damage to the developing brain. It represents a heterogeneous group of motor impairments [1], and often accompanies sensory, cognitive, intelligence, and communicative disabilities [2]. It is a severe childhood disorder associated with a very high disease burden. Regardless of the type and severity of CP, 52% of all patients suffer from mental illness [2]. Similarly, children with CP exhibit 3- to 4-fold higher internalized and externalized problems than the healthy population [3]. Among the neurodevelopmental disorders, Attention-Deficit Hyperactivity Disorder (ADHD) is known to manifest the highest comorbidity in CP [2]. Out of 90 Israel pediatric patients diagnosed with CP aged 1.8 to 15.4 years, 22.5% met the diagnostic criteria for ADHD [4]. Similarly, among 47 Norwegian children with CP, 24 were diagnosed with ADHD [5]. Nevertheless, ADHD symptoms are disregarded in CP patients, leading to delayed intervention.

ADHD is one of the most common neurodevelopmental disorders diagnosed in the field of child psychiatry, with a worldwide prevalence of approximately 3–10% [6]. It is a neurodevelopmental disorder characterized by persistent and developmentally inappropriate inattentiveness and hyperactivity/impulsivity [7]. These symptoms lead to substantial dysfunction in the home, social relationships and academics [8, 9]. Further, many children with ADHD show motor disturbances [10] and most of them display clumsy motor performance below the norms for their age [11].

Various studies have demonstrated that executive function of the cognitive domain in children with neurodevelopmental disorders are not fully developed during childhood. As a result, those with CP and ADHD appear to continuously experience difficulties in the academic fields and life than children with CP but without ADHD. To start with, both populations experience difficulties with sustained attention. The reduced processing speed and performance interferes with complex cognitive tasks that require cognitive and logical skills [12]. In fact, children with CP and ADHD exhibit slower processing speed compared to healthy peers [13]. They show selective attention deficits and impaired response and suppression to stimuli irrelevant to the task [14], so when the demands for information processing are high, they tend to exhibit poor performance. Further, they both perceive reduced levels of quality of life, with 75% of CP children expressing pain regardless of their gross movement and functional levels, which positively correlated with decreased quality of life [15, 16]. Due to consistent negative feedback under multiple settings [17], children diagnosed with CP or ADHD experience withdrawal and decreased quality of life.

As mentioned above, numerous studies have shown that the presence of concurrent CP and ADHD leads to the accumulation of negative experience, which may increase the risk of secondary psychological ailments [13, 15, 16]. As these conditions can be treated during childhood, the importance of early intervention is emphasized. Various therapeutic approaches, including cognitive behavioral therapy or medication to treat ADHD were adopted to those with CP or ADHD to alleviate the symptoms but showed restricted effects, which can be the limitation for these approaches on this particular population. Cognitive therapy reinforces the core network of brain associated with attention via systematic programs that improve cognitive functions, such as planning and information processing [4], even though a recent meta-analysis reported that cognitive therapy showed a significant improvement in visual and language working memory, it had no effect on suppression and inattentiveness. For this reason, it is recommended more as a supplementary treatment rather than a primary treatment for attention training [18]. Additionally, the fact that CP children are characterized by difficulties with fine motor skills, the positive effect of cognitive therapy that requires repetitive usage of computer programs may be minimal. Medication is one of the most widely used treatments. In a placebo-controlled study, patients with a dual diagnosis of CP and ADHD were treated with medication or placebo to investigate the effect on attention, and the results demonstrated short-term drug effect and minimal side effects, including loss of appetite or stomach aches [19]. However, some parents are cautious in administering medications to their children. Therefore, an alternative intervention appears to be necessary.

Recently, Equine-Assisted Activities and Therapies (EAAT) have received attention as it helps people suffering from a variety of psychological issues. Equine Assisted Activities and Therapies (EAAT) is a broad term that encompasses two main interventions; Equine Assisted Activities (EAA) and Equine Assisted Therapies (EAT). EAA includes instructions in riding skills, how to control the horse with verbal and nonverbal cues and horsemanship skills [20]. This intervention tends to emphasize activities that promoted social, cognitive, physical, psychological and social skills [20]. Earlier studies have adopted EAA in various fields of treatment. It inculcates a sense of self-efficacy in patients with psychiatric disabilities [21], and patients with stress or those who express irritability are more likely to experience a positive effect due to the physiologic response to the rhythmic repetitive movements of the horse [9, 22].

Although the effectiveness of EAA on attention or psychosocial functioning has been investigated [8, 9], studies have yet to evaluate the collective improvement in symptoms. Thus, the primary objective of this study was to investigate the effectiveness of EAA on attention in children with CP and their perceived quality of life. Moreover, the secondary objective was to evaluate the comorbidity between CP and ADHD and examine the effectiveness of EAA for those who have both CP and ADHD. We hypothesized that the EAA program would improve attention deficit/impulsivity and enhance patients’ quality of life. Also, we expected that CP accounts for some of the increased prevalence with ADHD.

## Methods

### Study participants

The study involved 47 CP outpatients aged 6 to 13 years who visited the Department of Rehabilitation within a university hospital located in Seoul from August 2017 to January 2019. The psychiatric diagnoses were based on the diagnostic criteria of the Diagnostic and Statistical Manual for Mental Disorders-5 (DSM-5) and confirmed the comorbidity by Kiddie Schedule for Affective Disorders and Schizophrenia Present and Lifetime Version (K-SADS-PL). The exclusion criteria were: patients with weight exceeding 35 kg, uncontrolled seizures; inability to perform one-step instruction due to visual and hearing impairment or intellectual disability; hip dislocation; scoliosis greater than 30°; less than 1-year history of musculoskeletal surgery; unrecovered fracture; botulinum toxin injection within 3 months; or riding that was inappropriate for other medical conditions.

The participants were randomly assigned to the EAA exercise group or the control group. The participants were assigned with trial ID numbers, created by a researcher not otherwise involved in the study. Prior to the 16-week study program, both groups participated in a pre-comprehensive assessment within 2 weeks, including intelligence, attention and psychological evaluation using Korean-Wechsler Intelligence Scale for Children-Fourth Edition (K-WISC-IV), Conner’s Continuous Performance Test (CPT), Korean ADHD Rating Scale (K-ARS) and Pediatric Quality of Life Inventory (PedsQl), which were used in previous studies to examine therapeutic effects on ADHD or CP children [9, 23]. 2-weeks after the program was completed, the post-test assessment, including attention and psychological evaluation was performed. Those who were assigned to the control group were given the opportunity to participate in the EAA program after the trial. All participants were fully informed about the study and signed the participant agreement form. One participant from the control group dropped out from the study before completion and the data was excluded for analysis. The study was approved by the Institutional Review Board of Samsung Medical Center (2017–06-045).

### Equine-assisted activities program (EAA)

Those included in the EAA exercise group participated in a 40-min class twice a week over the course of 16 weeks (total of 32 sessions). A single instructor conducted a group program for three children, and each child was assisted by a horse leader and a side walker. The instructor holds national therapeutic riding instructor certification and previously had obtained Professional Association of Therapeutic Horsemanship (PATH) registered therapeutic riding instructor certification. The sessions focused on mounted activities which consisted of exercises to facilitate accurate posture, balance and practice basic riding skills. The control group did not receive intervention and continued with their usual daily activities.

### Measurement tools

#### Korean-Wechsler intelligence scale for children-fourth edition (K-WISC-IV)

K-WISC-IV is a standardized measurement tool to assess intellectual function in children between ages 6 and 16 years and 11 months. The profile provides Full Scale Intelligence Quotient (FSIQ) for overall intellectual abilities and a total of five composite scores for specific cognitive abilities. K-WISC-IV showed high internal consistency and good test-retest correlation in previous studies [24].

#### Conner’s continuous performance test 2rd edition

This visual vigilance computerized test was developed to measure attention problems and has been widely used as a supplementary diagnostic tool for ADHD. The participants are instructed to respond to the computer screen by pressing the left button for every picture presented except the soccer ball. Based on the response, d’ (the accuracy in discriminating non-targets from targets), omission error (the number of times that the subject has failed to respond to the target), commission error (the number of times that the subject provided an incorrect response to the non-target), preservation error (the number of random responses, anticipatory response or a repeated response without consideration of the task requirements), mean response time (measured between the presentation of the target stimulus and a correct response), standard deviation of reaction time (variability or consistency of attention), and standard deviation of variable response time are calculated to determine index scores of attention and impulsivity. In the current study, all subjects underwent a 3- to 4-min practice session before starting the main evaluation. The test required a total of 30 min and the examiner remained in the room during the administration.

#### Korean ADHD rating scale (K-ARS)

The scale is used to assess ADHD symptoms in children. The score is based on each 9 items that reflect inattentiveness and hyperactivity/impulsivity, with a total of 18 items. Higher scores indicate increased attention impairment and impulse inhibition abilities. The Korean version of ARS was validated with reasonable internal consistency of 0.77–0.89 [25].

#### Pediatric quality of life inventory 4.0 genetic Core scale

The scale measures children’s general quality of life. It is composed of 21 items based on 4 dimensions: physical, emotional, social and academic aspects. Higher scores indicate higher quality of life. In this study, scores for each subscale were used to evaluate the treatment effect.

#### Kiddie schedule for affective disorders and schizophrenia present and lifetime version (K-SADS-PL)

K-SADS-PL was introduced by Kaufman and his colleges (1997) to assess current and past episodes of psychiatric disorders listed in Diagnostic and Statistical Manual of Mental Disorders-IV (DSM-IV). This semi-structured diagnostic instrument has been reported to be valid for the screening and diagnosis of childhood and adolescent disorders. The Korean Version of K-SADS-PL was translated and validated by Kim and his colleagues (2004) who reported excellent reliability and validity. In the present study, the tool was used to confirm the diagnoses of any existing psychiatric diagnosis.

### Statistical analysis

The demographic data of 46 participants (age, sex, and FSIQ) were compared via independent *t-*test. Repeated measures of analysis of variance (repeated ANOVA) were used to compare the overall improvement in attention and quality of life before and after treatment in the exercise and control group. The secondary analysis for intra-group comparison among subjects who were diagnosed with ADHD was based on t-test. A *p*-value less than 0.05 was considered statistically significant and results were analyzed using SPSS ver. 24 (IBM Corp., Armonk, NY, USA).

## Results

### General characteristics of the participants

Twenty-three participants were assigned to the exercise group (12 males, 11 females) and the control group (11 males, 12 females). The average age of participants in the exercise and control group is 7.78 ± 1.68 years, 7.30 ± 1.61 years, respectively. The average FSIQ for the exercise and control group is 79.91 ± 19.86, 79.70 ± 16.03, respectively. There was no group difference in demographic characteristics of sex or age and intelligence. Participant characteristics are summarized in Table 1. Fifteen children (31.91%) from the entire sample met the diagnostic criteria for ADHD. No other psychiatric diagnosis was made. The descriptive characteristics of the subsample are presented in Table 2.

**Table 1:** Description Characteristics of the Total Sample (*N* = 46)

Items	Exercise (*N* = 23)		Control (*N* = 23)		*p-*value†
	N	M ± SD	N	M ± SD	
**Sex**					.774
Males	12		11		
Female	11		12		
Age of years		7.78 ± 1.68		7.30 ± 1.61	.329
**K-WISC-IV**					
FSIQ		79.91 ± 19.86		79.70 ± 16.03	.266
VCI		92.13 ± 12.46		92.13 ± 12.46	
PRI		78.48 ± 17.87		78.48 ± 17.87	
WMI		83.91 ± 18.70		83.91 ± 18.70	
PSI		78.74 ± 19.21		78.74 ± 19.21	
**Comorbidity**					
**ADHD**					
Combined		4		3	
Inattentive		2		1	
Hyperactive/Impulsive		0		0	
NOS		2		3	

*Note.* †: Independent t-test. *ADHD* Attention Deficit/Hyperactivity Disorder, *NOS* Not Otherwise Specified, *M* Mean, *SD* Standard Deviation, *N* Number, *K-WICS-VI* Wechsler Intelligence Scale for Children 4th edition, *FSIQ* Full Scale Intelligence Quotient

**Table 2:** Description Characteristics of the Sub-Sample (*N* = 15)

	Exercise (*N* = 8)		Control group (*N* = 7)		*p*-value†
	N	M ± SD	N	M ± SD	
b S **Sex**					**.000**
Male	5		7		
Female	3		0		
**Age**		7.50 ± .926		7.14 ± 1.86	.204
**FSIQ**		74.50 ± 16.45		69.43 ± 11.27	.239

*Note.* †: Independent t-test. FSIQ: Full Scale Intelligence Quotient

### Effects of interventions on attention and quality of life

Analyzed by repeated measures of ANOVA, the perseveration error score decreased in a time-dependent manner (F = 12.34, *p* < .001). Group differences existed (F = 5.48, *p* < .05), representing a difference in effectiveness between groups. The change in mean perseveration score showed a significant decrease pre- and post-treatment from 61.30 to 53.04 in the exercise group; 51.57 to 49.91 in the control group. The d’ (*p* < .001), commission (*p* < .001), and hit reaction time standard deviation (*p* < .05) decreased in a time-dependent manner, but the group difference was insignificant.

The effects on quality of life suggest that physical (*p* = .000), emotional (*p* = .011), social (*p* = .000), and academic (*p* = .024) subdomains significantly increased in a time-dependent manner, but no group difference existed. The above results are presented in Table 3.

**Table 3:** Comparison of the Baseline and Post-Treatment CPT, ARS and PedsQL scores in Total Subjects

	Exercise (*N* = 23)		Control (*N* = 23)		*p-*value†	
	Baseline	Post-	Baseline	Post-		
**CPT**						
d’	59.00 ± 7.62	52.57 ± 10.23	54.00 ± 7.76	49.61 ± 8.11	time time*group	**.000** .349
Omission	71.70 ± 16.66	67.13 ± 18.61	59.91 ± 11.29	57.91 ± 14.84	time time*group	.246 .648
Comission	48.74 ± 9.90	42.96 ± 7.50	46.65 ± 9.47	43.13 ± 7.24	time time*group	**.000** .354
Perseveration	61.30 ± 13.65	53.04 ± 8.22	51.57 ± 8.77	49.91 ± 6.82	time time *group	**.001** **.024**
Hrt	69.87 ± 14.75	70.04 ± 14.22	65.04 ± 11.31	65.70 ± 12.31	time time*group	.766 .215
Hrt sd	65.74 ± 12.08	60.78 ± 12.14	55.83 ± 10.18	55.04 ± 9.77	time time*group	**.040** .131
**ARS**						
Parents_Total	12.22 ± 8.66	11.17 ± 8.32	11.13 ± 8.14	10.26 ± 8.20	time time*group	.138 .891
Parents_Inatten	7.70 ± 4.90	7.13 ± 5.22	6.35 ± 4.43	6.22 ± 5.03	time time*group	.384 .585
Parents_Hyp	4.52 ± 4.39	4.04 ± 3.70	4.78 ± 4.09	4.04 ± 3.51	time time*group	.090 .712
**PedsQL**						
Physical	60.73 ± 19.21	69.70 ± 15.81	65.90 ± 18.15	77.85 ± 15.10	time time*group	**.000** .568
Emotional	70.22 ± 24.84	79.13 ± 20.49	69.57 ± 27.67	82.17 ± 16.98	time time*group	**.011** .210
Social	58.48 ± 28.14	74.13 ± 16.90	61.30 ± 27.19	80.65 ± 22.02	time time*group	**.000** .253
Academics	69.35 ± 22.98	74.13 ± 18.99	72.39 ± 24.67	85.44 ± 12.33	time time*group	**.024** .284

*Note.* †: Repeated ANOVA. *CPT* Conner’s Continuous Performance Test, *Hrt* Hit Reaction Time, *Hrt Sd* Hit Reaction Time Standard Deviation, *PedsQL* Quality of Life 4.0 Genetic Core Scale, *ARS* ADHD Rating Scale, *Inatten* Inattention, *Hyp* Hyperactivity

### Effects of intervention on attention and quality of life in the ADHD subsample

A secondary analysis was conducted with 15 CP children who were diagnosed with ADHD. For attention, in the exercise group the change of d’ (*t = 2.713*, *p =* <.05), commission (*t =* 2.499, *p =* <*.*05), and perseveration score (*t =* 2.361, *p =* <.05) showed a significant difference during the 16-week program. Further, omission, hit reaction time, and hit block change showed a gradual improvement but was not statistically significant. In the control group, d’, omission and hit reaction time showed a decrease but was not significant. In the parent’s ARS self-report, the scores showed a decrease in both groups but without any significance.

In the case of quality of life, in the exercise group the change in social function (*t =* − 2.48, *p =* < .05) showed a significant increase after the intervention. Although the results were insignificant, the domains of physical and emotional functioning showed an increase whereas academic performance decreased. In the control group, physical, emotional, social functioning, and academic performance showed an increase, but was statistically insignificant. The above results are presented in Table 4.

**Table 4:** Comparison of the Baseline and Post-Treatment CPT, ARS and PedsQL scores in ADHD Subsample

	Exercise (*N* = 8)			***p†***	Control (*N* = 7)			***p†***
	Baseline	Post-	***t***		Baseline	Post-	***t***	
**CPT**								
d’	65.20 ± 7.01	57.88 ± 8.64	2.713	**.030**	55.00 ± 10.18	50.29 ± 7.11	1.639	.152
Omission	75.25 ± 12.68	70.75 ± 16.86	.717	.497	63.00 ± 14.41	57.71 ± 17.64	.864	.421
Commission	54.00 ± 8.78	46.75 ± 8.45	2.499	**.041**	47.71 ± 7.91	57.71 ± 17.64	−1.703	.280
Perseveration	70.63 ± 13.49	58.13 ± 7.28	2.361	**.050**	49.14 ± 5.08	50.86 ± 7.73	−.667	.530
Hrt	75.75 ± 13.44	68.75 ± 14.68	1.294	.237	64.29 ± 10.06	64.00 ± 17.49	.081	.938
Hrt sd	66.25 ± 20.70	62.88 ± 10.83	.661	.530	55.14 ± 14.42	57.86 ± 11.28	−.622	.557
**ARS**								
Parents_Total	21.25 ± 5.39	18.13 ± 5.84	1.89	.100	18.57 ± 8.12	15.57 ± 5.97	2.03	.089
Parents_Inatten	12.63 ± 2.92	10.88 ± 4.12	1.47	.185	10.29 ± 3.45	9.57 ± 2.88	1.00	.356
Parents_Hyp	8.63 ± 3.70	7.25 ± 2.82	1.72	.130	8.29 ± 4.99	6.00 ± 3.46	2.36	.056
**PedsQL**								
Physical	63.60 ± 14.95	76.86 ± 24.50	−1.93	.095	64.82 ± 22.27	78.30 ± 20.50	−1.721	. 136
Emotional	68.75 ± 14.33	80.25 ± 12.02	−1.58	.159	67.86 ± 33.65	84.71 ± 15.81	−1.085	.320
Social	55.00 ± 26.59	75.63 ± 22.41	**−**2.48	**.042**	67.86 ± 27.35	85.86 ± 13.86	−1.387	.215
Academics	74.73 ± 20.74	70.75 ± 18.38	0.36	.728	66.77 ± 33.39	84.29 ± 17.56	−1.067	.327

*Note*. †: Paired t-test. *CPT* Conner’s Continuous Performance Test, *Hrt* Hit Reaction Time, *Hrt Sd* Hit Reaction Time Standard Deviation, *PedsQL* Quality of Life 4.0 Genetic Core Scale, *ARS* ADHD Rating Scale, *Inatten* Inattention, Hyp: Hyperactivity

## Discussion

Previous studies that applied EAA to children with CP or ADHD evaluated the changes in CP and ADHD separately in attention, social problems, and motor function but did not consider symptomatic improvements collectively or the comorbidity of these disorders. Therefore, the primary purpose of the current study was to evaluate the therapeutic effect of 16-week EAA on attention and psychosocial functioning in children with CP. The secondary purpose was to confirm the comorbidity between CP and ADHD and examine the effectiveness of EAA for those who have both CP and ADHD.

The effects of EAA program on attention and quality of life in CP children are as follows. Participants in the EAA exercise group showed an improvement in attention. Specifically, their ability to distinguish target from non-target stimulus was enhanced, suggesting that the ability for sustained attention and suppression of impulse to unrelated stimulus was improved by the intervention. Our results are consistent with previously studies reporting that sustained visual and auditory attention increased post-computerized EAA in children with ADHD [26]. These findings suggest that the efforts to focus on the horse lead to improvements in sustained attention. However, it is important to note the discrepancy between the results of CPT and ARS based on the physical conditions of CP children. CP children exhibit a wide range of behavioral problems including physical activity, neuromuscular dysfunction, and muscle weakness [27], and events extending to the presence of epilepsy, limited or defective walking ability and severe pain [28]. Due to these physical conditions, it is strongly likely that the caregivers fail to notice the inattentiveness or hyperactivity, as the ARS hyperactivity score was reported in the low range.

A secondary analysis of a subsample of CP children who were diagnosed with ADHD was performed. The overall function of the ADHD group performing exercise showed a greater improvement than the control group with ADHD. More specifically, most of the indices of attention showed an increase. The sub-domain of social functioning in quality of life showed an increase. Consistent with earlier studies, the social skills of disabled children improved significantly after an eight-week EAA program, whereas the control group revealed no significant differences [29]. This result is meaningful because among the sub-domains of quality of life, social functioning is known to be associated with the most dysfunction in this particular population. Compared to healthy peers, those with CP were correlated with low levels of social adaptation, including absence of close friends, loneliness and experience of being bullied [2]. Similarly, 55.3% of 66 parents of children with CP reported peer problems [29]. Further, children with CP have low social engagement and relationships and are at an increased risk of being bullied [30]. In a similar context, ADHD children not only fail to have peer relationships due to lack of patience and high levels of distractibility but also disrupt others with unnecessary hyperactivity [31]. Furthermore, since ADHD children show multiple problematic behaviors, actions before considering others’ feelings and lack of the ability to control their urge in social situations [32], they are at a higher risk of being rejected by peers. In addition, those with ADHD symptoms do not respond appropriately to social cues during interpersonal interactions due to social immaturity [31]. Altogether, these negative experiences lead to difficulties in social participation. Therefore, the benefit of EAA for psychosocial improvement is greatly enhanced.

The broader symptomatic improvement in attentional and psychosocial aspects of the CP and ADHD comorbidity groups may suggest that EAA is highly appropriate for patients with neurodevelopmental disorders. In fact, in the current study 31.91% of CP patients were diagnosed with ADHD. It is essential to scrutinize the ADHD symptoms of CP patients under clinical settings. As the coexistence of the disease is increased, the poor adaptability interferes with functional ability for activities of daily life, underscoring the importance of early intervention in these children.

The mechanism of EAA in improving attention and social skills is uncertain, but there are several possible explanations. One of the possible hypotheses is that during the repetitive and rhythmic movements of the horse in EAA, every participant begins to anticipate every movement. This practice leads to modification and reorganization of multiple systems in the brain including the sensory, muscular and limbic systems [33], which eventually enhance the inhibitory, cognitive, memory and attention domains of function. Further, the therapeutic effect promotes self-regulation in which riding a horse enables development of problem-solving skills in children and a sense of control over their impulsivity by mastering direction and speed [34]. A few studies have demonstrated that the interactive experience with the horses enhance the motivation to learn [35]. Moreover, an earlier study has been published that EAA in ADHD could modulate functional connectivity in the regions related to default mode network and behavioral inhibition system relating with ADHD symptom improvement [9].

A positive effect on social maladaptation may occur via interactions with the horses. The behavioral response and social structure of horses is similar to that of humans, which provides an opportunity to simulate relationships in a non-threatening environment [9, 36]. Also, nonverbal communication and joint attention is promoted by interaction with horses [23] encouraging children to learn and control their emotions via rapport with the horse. In fact, a previous meta-analysis of EAA found a moderate effect in improving social skills [37].

The strength of this study is that unlike previous studies, an objective measurement tool of CPT was used to evaluate attention levels. In most previous studies, the assessment of psychological symptoms in early childhood relied heavily on caregivers or teachers’ response obtained through self-reports [38]. This approach may lead to lack of symptom validation. As a result, the importance of laboratory or objective evaluation to measure sustained attention and vigilance are being increasingly emphasized [38]. Compared with other laboratory tools, CPT is a standardized and computerized tool, which is easily accessible, norm-referenced and thus relatively inexpensive to develop, and easy to score [39].

The limitations are as follows. First, although participants were randomly assigned to each group the baseline CPT scores showed a significant difference. However, after the EAA exercise group showed a radical increase in attention improvement post-treatment compared to the control group, it could be interpreted as therapeutic effect. Second, this study was restricted to a small sample of outpatients in one particular hospital, and therefore the findings may not be generalized to other CP patients. Additional studies with larger samples are needed to replicate the findings and determine whether the results can be generalized across CP outpatients. Lastly, the current study did not examine the long-term therapeutic effect, so maintenance of the effect was not shown. Further studies that include a follow-up session after a certain period a time seems meaningful.

## Conclusion

In conclusion, to the best of our knowledge, this is the first study examining the clinical effects of EAA using the objective measurement in the group with both CP and ADHD for improving attention and social skills. For the CP population, the exercise group showed an enhanced attention, establishing the effectiveness of EAA. Furthermore, it seems to be more effective when CP and ADHD coexists, as both attention and social skills improved. Lastly, our study confirmed a high comorbidity of CP and ADHD. Based on these results, EAA can be considered as a non-pharmacological option to treat attentional problems for children with CP, and especially those with both CP and ADHD. Further epidemiological investigations in this area are certainly warranted for further understanding.

## Data Availability

The datasets used and/or analyzed during the current study are available from the corresponding author on reasonable request.

## Abbreviations

CP: Cerebral Palsy
ADHD: Attention-Deficit/Hyperactivity Disorder
EAA: Equine-Assisted Activities
DSM-5: Diagnostic and Statistical Manual for Mental Disorders, 5th edition
K-SADS-PL: Kiddie Schedule for Affective Disorders and Schizophrenia Present and Lifetime Version
K-WISC-IV: Korean-Wechsler Intelligence Scale for Children, 4th edition
FSIQ: Full Scale Intelligence Quotient
CPT: CPT: Conner’s Continuous Performance Test, 2nd Edition
K-ARS: Korean ADHD Rating Scale
PedsQL: Pediatric Quality of Life Inventory 4.0 Genetic Core Scale
ANOVA: Analysis of variance analysis
